# C-Reactive Protein and Hemogram Parameters for the Non-Sepsis Systemic Inflammatory Response Syndrome and Sepsis: What Do They Mean?

**DOI:** 10.1371/journal.pone.0148699

**Published:** 2016-02-10

**Authors:** Bulent Gucyetmez, Hakan K. Atalan

**Affiliations:** 1 Department of Anesthesiology, Acıbadem University Faculty of Medicine, Istanbul, Turkey; 2 Intensive Care Unit, Ataşehir Memorial Hospital, Istanbul, Turkey; University of Leicester, UNITED KINGDOM

## Abstract

**Objectives:**

Sepsis is one of the most common reasons of increased mortality and morbidity in the intensive care unit. The changes in CRP levels and hemogram parameters and their combinations may help to distinguish sepsis from non-sepsis SIRS. The aim of this study is to investigate the CRP and hemogram parameters as an indicator of sepsis.

**Methods:**

A total of 2777 patients admitted to the ICU of two centers between 2006–2013 were evaluated retrospectively. The patients were diagnosed as SIRS (-), non-sepsis SIRS and sepsis. The patients who were under 18 years old, re-admitted, diagnosed with hematological disease, on corticosteroid and immunosuppressive therapy, SIRS (-), culture negative, undocumented laboratory values and outcomes were excluded. 1257 patients were divided into 2 groups as non-sepsis SIRS and sepsis. The patients’ demographic data, CRP levels, hemogram parameters, length of ICU stay and mortality were recorded.

**Results:**

1257 patients were categorized as non-sepsis SIRS (816, 64.9%) and sepsis (441, 35.1%). In the multivariate analysis, the likelihood of sepsis was increased 3.2 (2.2–4.6), 1.7 (1.2–2.4), 1.6 (1.2–2.1), 2.3 (1.4–3.8), 1.5 (1.1–2.1) times by the APACHE II≥13, SOFA score≥4, CRP≥4.0, Lym_C_<0.45 and PLT_C_<150 respectively (p<0.001 p = 0.007 p = 0.004 p<0.001 p = 0.027). The likelihood of sepsis was increased 18.1 (8.4–38.7) times by the combination of CRP≥4.0, lym_C_<0.45 and PLT_C_<150 (P<0.001).

**Conclusions:**

While WBC_C_, Neu_C_, Neu%, NLCR and Eo_C_ are far from being the indicators to distinguish sepsis from non-sepsis SIRS, the combinations of CRP, Lym_C_ and PLT_C_ can be used to determine the likelihood of sepsis.

## Introduction

Systemic inflammatory response syndrome (SIRS) which occurs due to infection or non-infectious reasons is a clinical status. SIRS is the occurrence of at least two of the following criterias: fever>38°C or <36°C, heart rate>90 min^-1^, respiratory rate >20 min^-1^, white blood cell count (WBC_C_)>12000 or >4000 L^-1^ [1]. In the last guideline, SIRS criterias are diagnostic criteria for sepsis [2]. However, at the ICU admission, the patients often display SIRS criterion but sepsis is not diagnosed in a considerable number of these patients. It is known that sepsis is one of the most common reasons of increased mortality and morbidity in the intensive care unit (ICU) [3]. Therefore, it is crucial to distinguish sepsis from non-sepsis SIRS at the ICU admission. C-reactive protein (CRP) which is produced in liver is an acute phase reactant and it is known that CRP is comprised of five subunits and deposited at sites of inflammation [4]. In the last guideline, increase in CRP levels by 2 standard deviation (SD) is defined as a diagnostic criteria for sepsis [2]. However, CRP level can be increased by other factors such as cardiovascular disease, chronic obstructive pulmonary disease and obstructive sleep apnea syndrome [4–7]. Furthermore, the increase in CRP levels by 2 SD is commonly seen in a considerable number of patients admitted to the ICU. Hence, hemogram parameters which are inexpensive laboratory tests can be helpful for diagnosis of sepsis. Although WBC_C_ was indicated as a sepsis criteria in the last guideline, some studies have demonstrated that it has low sensitivity and specificity for sepsis diagnosis [8,9]. Neutrophil count (Neu_C_) and eosinophil count (Eo_C_) were used as a predictor of sepsis in the early 1990s [9–13]. Eo_C_ and lymphocyte count (Lym_C_) were known to decrease in acute stress disorders such as trauma or infection [14,15]. Thus, in some studies, Eo_c_, Lym_C_ and neutrophil-lymphocyte count ratio (NLCR) were used as indicators for sepsis diagnosis [8,9,16,17]. The changes of CRP levels and hemogram parameters and their combinations may help to distinguish sepsis from non-sepsis SIRS at the ICU admission. The aim of this study was to investigate the CRP and hemogram parameters as an indicator of sepsis diagnosis.

## Materials and Methods

### Study design

**Fig 1:**
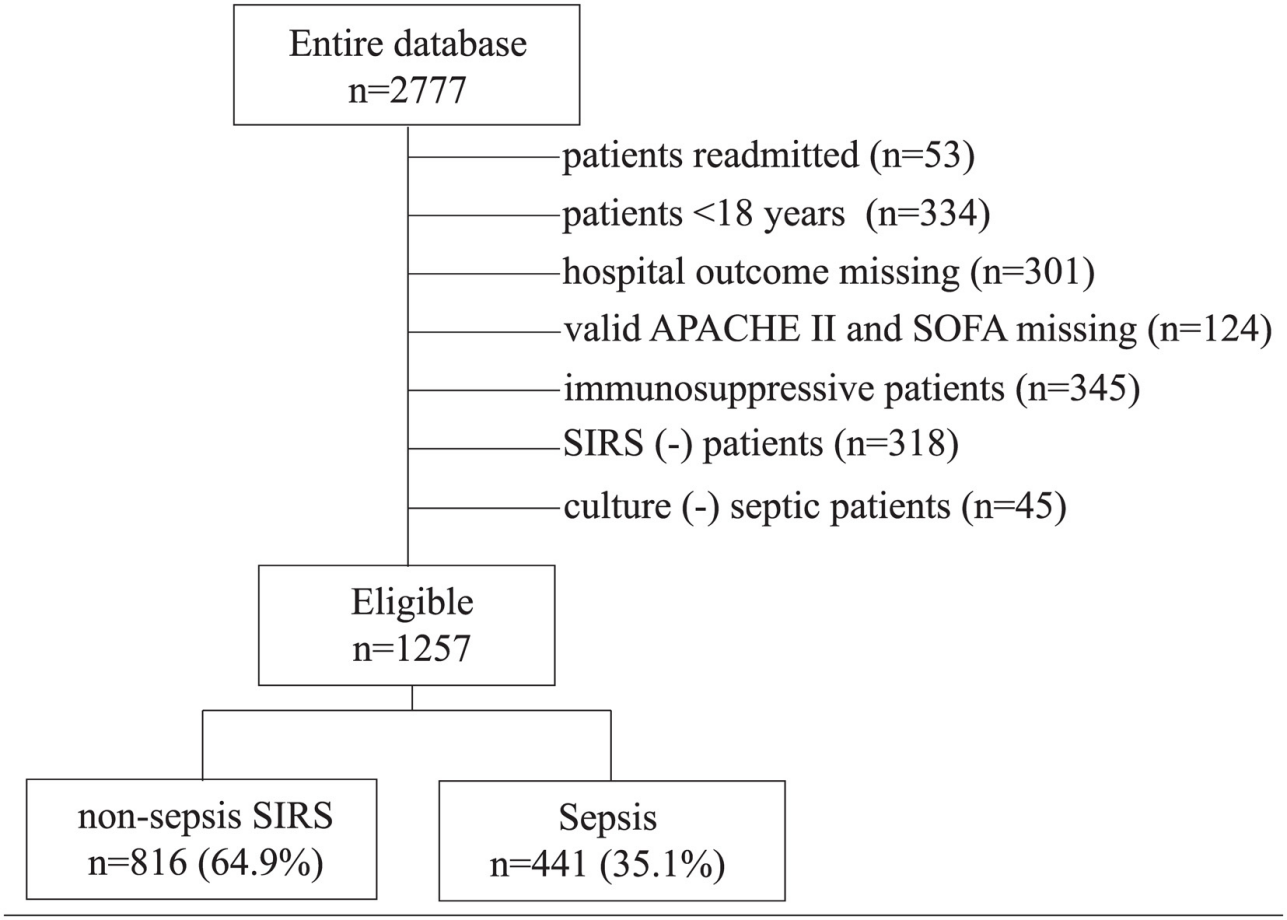
Study flowchart. Abbreviations: APACHE II, Acute Physiology And Chronic Health Evaluation; SIRS, systemic inflammatory response syndrome; SOFA, Sequential Organ Failure Assessment.

A total of 2777 medical and surgical patients admitted to the ICU’s of Acibadem International Hospital and Ataşehir Memorial Hospital between 1 January 2006 and 31 December 2013 were evaluated retrospectively. The study protocol was approved by the Acibadem University Medical Faculty Ethics Committee. Informed consent was not required because of the retrospective nature of the study. In the process of evaluating files of patients, the personal details of these patients were not recorded. The patients were diagnosed as SIRS (-), non-sepsis SIRS and sepsis at the ICU admission. SIRS and sepsis were defined in accordance with 1992 Sepsis Guideline [1]. The patients who were under 18 years old, re-admitted, diagnosed with hematological disease, on corticosteroid and immunosuppressive therapy, SIRS (-), culture negative, undocumented laboratory values and outcomes were excluded. The eligible patients were divided into 2 groups namely non-sepsis SIRS and sepsis (Fig 1).

### Database

The patients’ age, gender, APACHE II (Acute Physiology And Chronic Health Evaluation) and SOFA (Sequential Organ Failure Assessment) scores, diagnosis (medical, elective and emergency surgery), length of ICU stay, mortality, CRP (mg dL^-1^), WBC_C_ (x10^3^ μL^-1^), Neu_C_ (x10^3^ μL^-1^), Lym_C_ (x10^3^ μL^-1^), NLCR, Eo_C_ (μL^-1^), platelet count (PLT_C_) (x10^3^ ul^-1^), mean platelet volume (MPV) (fL) were recorded. All laboratory values were obtained from the Acibadem Inernational Hospital and Ataşehir Memorial Hospital databases.

### Sepsis Definition

Sepsis was defined in accordance with 1992 sepsis guideline [1]. The patients who had at least two SIRS criterias (WBC_C_>12.000 or <4000 or >10% immature form; temperature>38.3°C or <36.0°C; respiratory rate >25 or PaCO_2_<32 mmHg; heart rate >90) on ICU admission and positive culture were considered to be sepsis. CRP was not used as a criterion in the diagnosis of sepsis.

### Laboratory measurements

Evaluated blood samples had been taken at ICU admission prior to any medical treatment. In both hospitals, all blood samples taken for hemogram parameters were stored in the tubes in which ethylene diamine tetra acetic acid was used as anticoagulant and the measurements were carried out with Sysmex hematology analyzer (Sysmex XT-2000i, Kobe, Japan). WBC_C_, Neu_C_, Lym_C_ and Eo_C_ were measured by the application of semiconductor flow cytometry method; PLT_C_ was measured with hydrodynamic focusing DC detection and semiconductor laser flow cytometry method and MPV was measured with the use of PLT-particle-size distribution method. The blood samples taken for CRP was stored in vacuumed tubes in which silica gel was used. CRP was measured with a Cobas Integra (Roche Diagnostics, Mannheim, Germany) device by applying the immunoturbidimetry method.

### Cultures

The patients’ cultures (bloodstream, respiratory secretion, urine, cerebrospinal fluid) which had been taken at the ICU admission before antibiotics were administered were recorded. Colony counts 100000 CFU mL^-1^ or more were accepted as positive culture. The type of microorganisms were recorded as gram-negative bacteria, gram-positive bacteria, fungi and multiple microorganisms. There was no viremia in any patients.

### Statistical analysis

The stastistical analysis was perfomed using the Wizard Pro Version 1.7.20 (154). All variables in the database were summarized using descriptive statistics. Categorical data were described with number (percentage) and analyzed with chi-square test. Sepsis and non-sepsis SIRS groups and survival and non-survival groups were compared with Mann Whitney U test due to non-normal distribution patterns. Results were given as percentage and median (interquartiles). Effects of parameters to estimate sepsis were evaluated with multivariate logistic regression model. Logistic regression analysis model included age, APACHE II and SOFA scores, diagnosis at ICU admission, CRP, WBC_C_, Lym_C_, NLCR, Neu_C_, PLT_C_. Cut-off values for sepsis were determined by using the received operation curve (ROC) analysis. Type 1 error level was set as 5%. Correlation test was used for correlation between parameters and given as r^2^ value.

## Results

**Fig 2:**
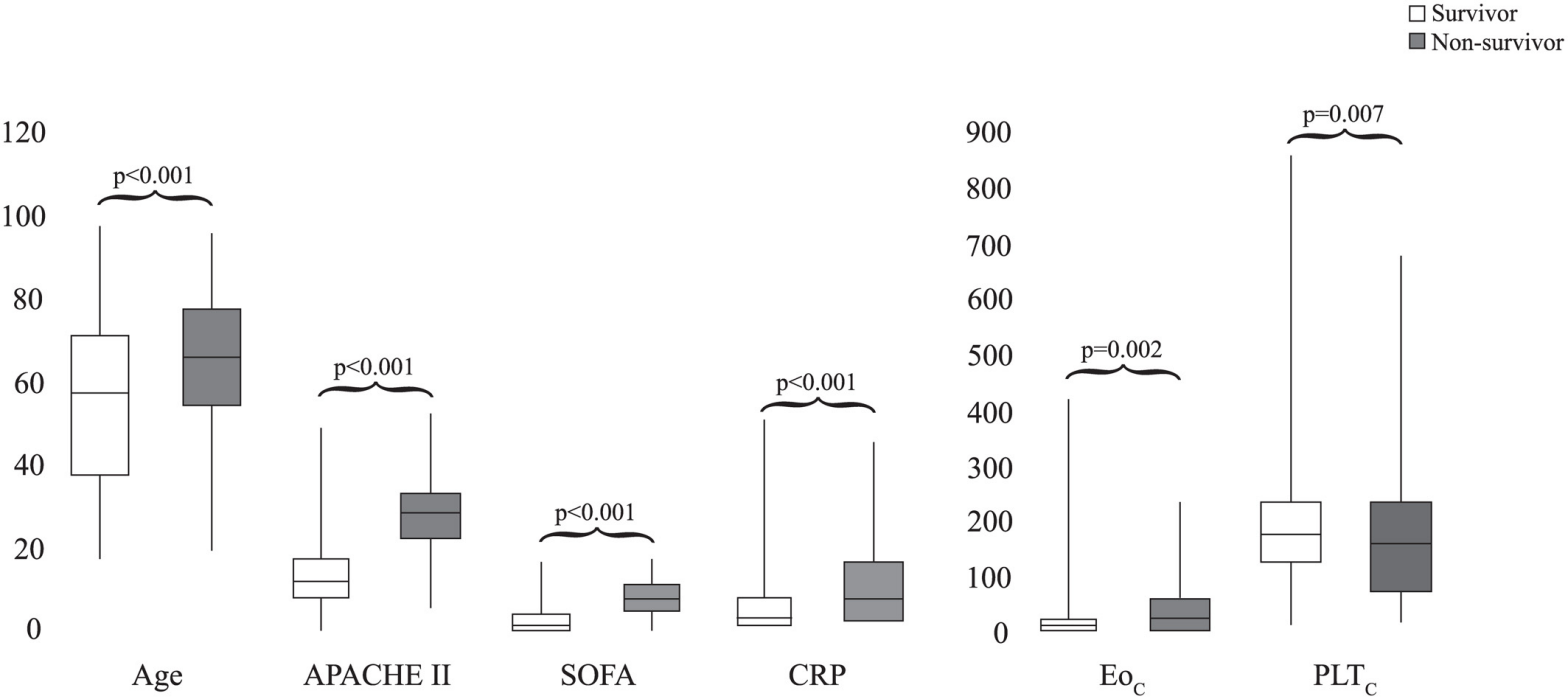
Comparison of survivor and non-survivor patients. Abbreviations: APACHE II, Acute Physiology And Chronic Health Evaluation; CRP, c-reactive protein; Eo_C_, eosinophil count; PLT_C_, platelet count; SOFA, Sequential Organ Failure Assessment.

1257 patients were included in the study. Non-sepsis SIRS group consisted of 816 (64.9%), sepsis group consisted of 441 (35.1%) patients (Fig 1). In the sepsis group; age, APACHE II and SOFA scores, length of ICU stay, mortality, CRP and NLCR were significantly higher than non-sepsis SIRS group (p<0.001 for each). WBC_C_, Neu_C_, Lym_C_ and PLT were significantly lower in sepsis group than non-sepsis SIRS group (p = 0.003 p = 0.005 p<0.001 p = 0.01 respectively). Gender, Neu%, Eo_C_ and MPV were similar in both groups (p = 0.906 p = 0.312 p = 0.176 p = 0.733 respectively). Gram-negative microorganisms were most common in the sepsis group (28.1%). Cut off values of CRP, Lym_C_, Neu_C_, NLCR and PLT_C_ for sepsis were ≥4.0, <0.45, ≥10.0, ≥14.2 and <150 (Table 1). In non-survivor patients, age, APACHE II and SOFA scores, CRP and Eo_C_ were significantly higher; PLT_C_ was significantly lower than survivor patients. (p<0.001 p<0.001 p<0.001 p<0.001 p = 0.002 and p = 0.007) (Fig 2).

**Table 1:** Demografic data and clinicall outcome.

	non-sepsis SIRS (n = 816)	Sepsis (n = 441)	p
Age, years,	55 (37–69)	63 (51–76)	*<0*.*001*
Male, n (%)	482 (59.1)	262 (59.4)	0.906
APACHE II	9 (6–13)	18 (14–25)	*<0*.*001*
SOFA	1 (0–2)	4 (1–7)	*<0*.*001*
Diagnosis			*<0*.*001*
Elective surgery, n (%)	540 (66.3)	76 (17.2)	*<0*.*001*
Medical diseases, n (%)	240 (29.4)	354 (80.3)	*<0*.*001*
Emergency surgery, n (%)	36 (4.3)	11 (2.5)	*<0*.*001*
Microorganisms n (%)	0 (0.0)	124 (28.1)	*<0*.*001*
Gram-negative	0 (0.0)	64 (14.5)	*<0*.*001*
Gram-positive	0 (0.0)	86 (19.5)	*<0*.*001*
Fungi	0 (0.0)	167 (37.9)	*<0*.*001*
Multiple organism			*<0*.*001*
Length of ICU stay, days	1 (1–2)	4 (2–10)	*<0*.*001*
Mortality, n(%)	25 (3.1)	104 (23.6)	*<0*.*001*
CRP, (<0.5)^a^, (≥4.0)^b^	2.0 (0.5–6.1)	5.6 (1.6–13.9)	*<0*.*001*
WBC_C_, (3.98–10.04)^a^	11.27 (8.18–15.05)	10.04 (7.1–14.62)	*0*.*003*
Neu_C_, (1.56–6.13)^a^, (≥10.0)^b^	9.29 (6.55–12.7)	8.27 (5.58–12.73)	*0*.*005*
Neu (%)	85 (80–88)	85 (79–90)	0.312
Lym_C_, (1.18–3.74)^a^, (<0.45)^b^	0.93 (0.62–1.36)	0.71 (0.44–1.16)	*<0*.*001*
NLCR, (≥14.2)^b^	10 (6.7–14.5)	11.5 (7.2–18.6)	*<0*.*001*
Eo_C_, (40–360)^a^	10 (0–40)	10 (0–30)	0.176
PLT, (182–369)^a^, (<150)^b^	190 (133–242)	171 (101–256)	*0*.*01*
MPV, (9.4–12.4)^a^	10.1 (9.4–10.7)	10 (9.3–10.8)	0.733

^a^ normal values for hemogram parameters.

^b^ cut off values for likelihood of sepsis.

Results were given as percentage and median (interquartiles). Mann-Whitney U and chi-square tests were used for analysis. P<0.05 was accepted for statistically significant. Abbreviations: APACHE II, Acute Physiology And Chronic Health Evaluation; CRP, C-reactive protein; Eo_C_, eosinophil count; MPV, mean platelet volume; Neu_C_, neutrophil count; NLCR, neutrophil-lymphocyte count ratio; Lym_C_, lymphocyte count; PLT_C_, platelet count; SOFA, Sequential Organ Failure Assessment; WBC_C_, white blood cell count

**Fig 3:**
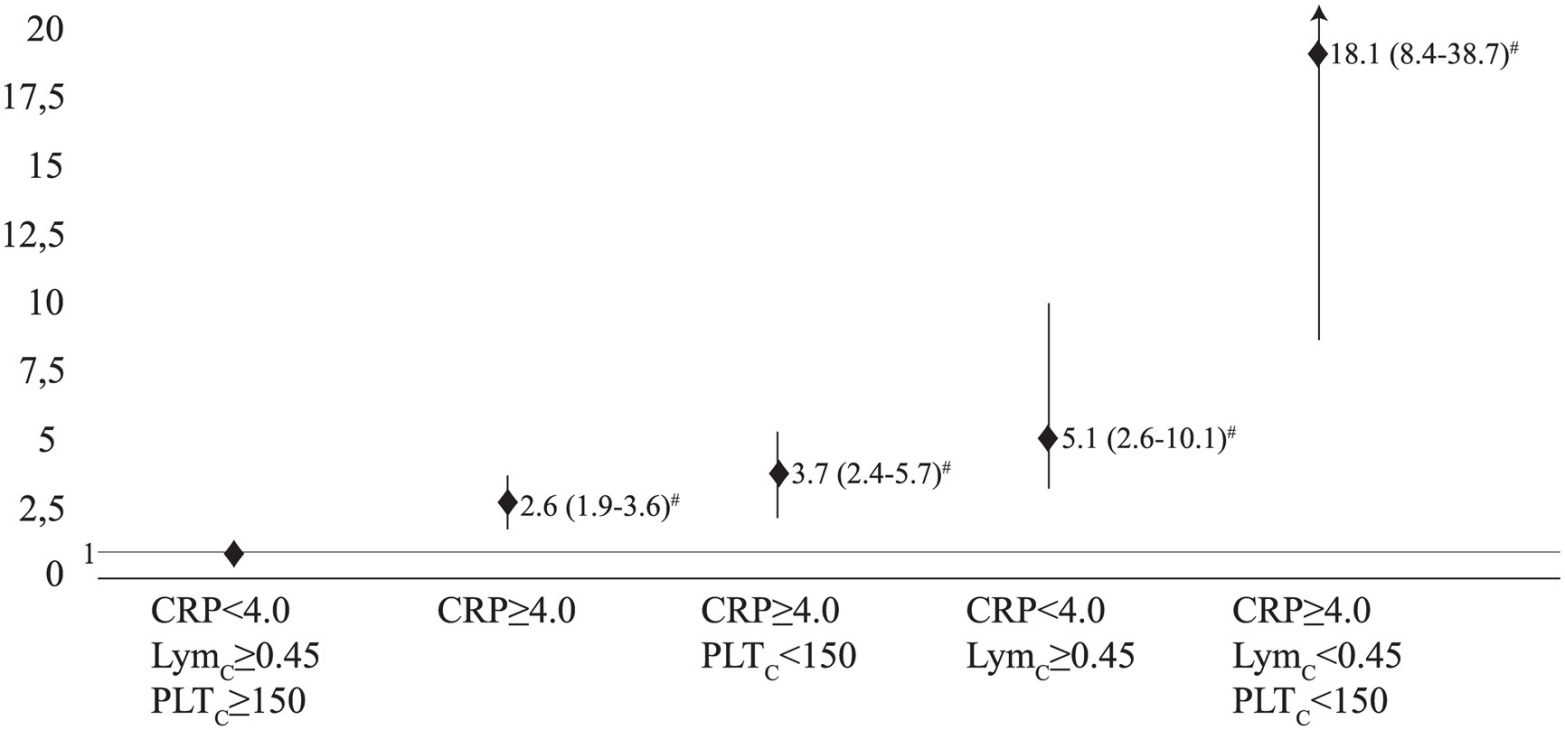
Combinations of CRP and hemogram parameters for likelihood of sepsis. Abbreviations: CRP, c-reactive protein; Eo_C_, eosinophil count; PLT_C_, platelet count. ^#^, p<0.001.

In the multivariate analysis, the likelihood of sepsis was increased 3.2 (2.2–4.6), 1.7 (1.2–2.4), 1.6 (1.2–2.1), 2.3 (1.4–3.8), 1.5 (1.1–2.1) times by the APACHE II≥13, SOFA score≥4, CRP≥4.0, Lym_C_<0.45 and PLT_C_<150 respectively (p<0.001 p = 0.007 p = 0.004 p<0.001 p = 0.027) (Table 2). The likelihood of sepsis was increased 18.1 (8.4–38.7) times by the combination of CRP≥4.0, lym_C_<0.45 and PLT_C_<150 (p<0.001) (Fig 3).

**Table 2:** Multivariate logistic regression model for sepsis.

	OR (95% CI)	p
Age	1.001 (0.993–1.009)	0.776
Medical disease	5.3 (3.7–7.7)	*<0*.*001*
APACHE II≥13	3.2 (2.2–4.6)	*<0*.*001*
SOFA score≥4	1.7 (1.2–2.4)	*0*.*007*
CRP≥4.0	1.6 (1.2–2.1)	*0*.*004*
WBC_C_<4.0	1.2 (0.6–2.4)	0.577
WBC_C_>12.0	(0.5–1.04)	0.083
Neu_C_≥10	1.1 (0.8–1.6)	0.630
Lym_C_<0.45	2.3 (1.4–3.8)	*<0*.*001*
NLCR≥14.2	1.4 (0.9–2.1)	0.142
PLT_C_<150	1.5 (1.1–2.1)	*0*.*027*

Abbreviations: 95% CI, 95% confidence interval; OR, odds ratio

## Discussion

The present study shows that CRP≥4.0, Lym_C_<0.45 and PLT_C_<150 at the ICU admission can be helpful in identifying sepsis. While WBC_C_, Neu_C_, Eo_C_ and Neu% do not have any contribution towards distinguishing sepsis from non-sepsis SIRS, combinations of CRP, Lym_C_ and PLT_C_ can be used to determining sepsis at the ICU admission.

CRP values over 0.5 mg dL^-1^ are shown to be related to the infection-induced inflammatory response [18]. Increase in CRP levels by 2 SD was also defined as sepsis criteria in 2012 sepsis guideline [2]. In the present study, 868 (69.1%) patients had got an increase in CRP levels by 2 SD at the ICU admission. However, 362 (41.7%) of them was diagnosed as sepsis. In 97 (21.7%) of septic patients, cardiovascular diseases and COPD were determined. Their median CRP level was 8. Furthermore, there was a poor positive correlation between CRP and each of age and sepsis (r^2^ = 0.04 and r^2^ = 0.09). Cardiovascular diseases and COPD are generally determined in overaged and it can be a reason for that correlation. 506 patients had got an increase in CRP levels by 2 SD but they were not diagnosed as sepsis. 344 (68%) of them was elective and emergency surgery patients. That results show that CRP is an inflammatory marker and it can be affected from many inflammatory clinical status. There are studies showing that procalcitonin (PCT) is a valuable marker compared to CRP as an indicator of infection. However, there are studies supporting the opposite findings [19–22].

Although CRP was related with sepsis and mortality in our patients, we are of the opinion that a combined evaluation of CRP and other hemogram parameters would increase the efficiency in diagnosing sepsis (Figs 2 and 3 and Table 2).

While <4000 or >12000 WBC_C_, was described as SIRS criterion in 1992 guideline, it was among the inflammatory variables of sepsis in 2012 guideline. [1,2]. Kim et al. did not indicate any difference in WBC_C_ of sepsis and non-sepsis groups [8]. However, de Jagger et al., showed that AUC value (0.53) of WBC_C_ for infection was not a more reliable marker than other hemogram parameters [9]. In our study, we found out that in sepsis group, WBC_C_ was significantly lower than non-sepsis SIRS group. We believe that this difference isn’t very important since median values of WBC_C_ for both groups are in normal range. Additionally, we didn’t find any relationship between WBC_C_ and each of CRP, sepsis and mortality. Although WBC_C_ is a diagnostic criteria for sepsis, we assume that WBC_C_ at the ICU admission is far from being an important marker in diagnosing sepsis.

In endotoxemia, it is known that Neu_C_ increases while Lym_C_ decreases in the circulation [23]. Hawkins et al. showed resistant B and T lymphopenia in gram-positive bacteraemia [24]. We indicated that there was no difference between CRP, Neu_C_ and Lym_C_ values of gram-negative and gram-positive groups. It was stated that NLCR was an indicator of infection [25]. de Jagger et al. argued that Lym_C_ was a good indicator for infection and they indicated that NLCR had higher AUC value for mortality but did not have significant importance in the multivariate analysis [9,26]. Although Terradas et al. detected NLCR increase in sepsis and did not evaluate the effect of Neu_C_ and Lym_C_ on this ratio [17]. In the present study, while Neu_C_ and Lmy_C_ were significantly decrease, NLCR was also significantly increase in sepsis group. In this respect, the reason of increased NLCR in sepsis group can be a greater decrease in Lym_C_ than Neu_C_. In multivariate analysis, the likelihood of sepsis was increased by only Lym_C_<0.45 (Table 2). We indicated that the likelihood of sepsis was increased by increased CRP with lymphopenia (Fig 3). For this reason, we strongly believe that Lym_C_ can be more helpful than Neu_C_ and NLCR for diagnosis of sepsis.

In acute infection, it is known that eosinopenia develops due to peripheral sequestration and suppression of mature eosinophil production and secretion from bone marrow [27]. Acute stress-related endogenous corticosteroid production or exogenous corticosteroid use may cause eosinopenia, as well [8]. In order to make a correct interpretation of Eo_C_, we excluded the patients on corticosteroid and other immunosuppressive agents. Terradas et al. indicated that increased Eo_C_ was an indicator of recovery and Eo_C_<50 was an indicator of bacteraemia [17]. Abidi et al. made the same conclusion for Eo_C_<40 [16]. However, there was no information about the patients on corticosteroid who were excluded in these two studies. On the other hand, Kim et al. excluded patients with corticosteroid therapy in pediatric patient group and showed that Eo_C_<15 increased the rate of mortality 2.96-fold [8]. Yet, they did not find out significant relationship between infection and Eo_C_. We found out similar Eo_C_ values in both groups (Table 1). Even in non-survivor patients, Eo_C_ was significantly higher than survivor patients (Fig 2). We can speculate that increased Eo_C_ in non-survivor patients may be due to relative adrenal insufficiency. Therefore, Eo_C_ was also far from being an important marker in diagnosing sepsis.

PLT_C_ was identified as a diagnostic criteria for sepsis in the last guideline [2]. In present study, we also found out that PLT_C_ related with sepsis and mortality (Tables 1 and 2 and Fig 2).

## Conclusions

CRP≥4.0, Lym_C_<0.45 and PLT_C_<150 can be used as indicators to distinguish sepsis from non-sepsis SIRS. Thus, the combinations of these markers can be more helpful to predict sepsis at the ICU admission. Even WBC_C_, Neu_C_, Neu%, NLCR and Eo_C_ are far from being the indicators to distinguish sepsis from non-sepsis SIRS.

## Supporting Information

S1 Text Dataset. (XLS)
